# Evaluating a 12-week aerobic exercise intervention in adults with persisting post-concussive symptoms

**DOI:** 10.3389/fneur.2024.1482266

**Published:** 2024-12-24

**Authors:** Leah J. Mercier, Samantha J. McIntosh, Chloe Boucher, Julie M. Joyce, Julia Batycky, Jean-Michel Galarneau, Joel S. Burma, Jonathan D. Smirl, Michael J. Esser, Kathryn J. Schneider, Sean P. Dukelow, Ashley D. Harris, Chantel T. Debert

**Affiliations:** ^1^Department of Clinical Neurosciences, Division of Physical Medicine and Rehabilitation, University of Calgary, Calgary, AB, Canada; ^2^Hotchkiss Brain Institute (HBI), University of Calgary, Calgary, AB, Canada; ^3^Department of Radiology, University of Calgary, Calgary, AB, Canada; ^4^Sport Injury Prevention Research Centre (SIPRC), Faculty of Kinesiology, University of Calgary, Calgary, AB, Canada; ^5^Libin Cardiovascular Institute of Alberta, University of Calgary, Calgary, AB, Canada; ^6^Alberta Children’s Research Institute (ACHRI), University of Calgary, Calgary, AB, Canada; ^7^Cerebrovascular Concussion Laboratory, Faculty of Kinesiology, University of Calgary, Calgary, AB, Canada; ^8^Department of Pediatrics, Section of Neurology, University of Calgary, Calgary, AB, Canada

**Keywords:** concussion, mild traumatic brain injury, aerobic exercise, quality of life, persisting post-concussive symptoms

## Abstract

**Background:**

Although guidelines support aerobic exercise in sub-acute mild traumatic brain injury (mTBI), evidence for adults with persisting post-concussive symptoms (PPCS) after mTBI is lacking. The objective was to evaluate the impact of a sub-symptom threshold aerobic exercise intervention on overall symptom burden and quality of life in adults with PPCS.

**Methods:**

This prospective cohort study was nested within the ACTBI Trial (Aerobic Exercise for treatment of Chronic symptoms following mild Traumatic Brain Injury). A total of 50 adults with a diagnosis of mTBI, PPCS and exercise intolerance completed a 12-week sub-symptom threshold aerobic exercise intervention either immediately after enrollment (i-AEP group; *n* = 27) or following 6-weeks of stretching (d-AEP group; *n* = 23). Data from all participants (*n* = 50) were included in the combined AEP (c-AEP) group. The primary outcome was symptom burden on the Rivermead Post Concussion Symptoms Questionnaire (RPQ). Secondary outcomes included measures of quality of life and specific post-concussive symptoms (depressive and anxiety symptoms, functional impact of headache, fatigue, sleep, dizziness and exercise tolerance). Heart rate, blood pressure and heart rate variability were also assessed to understand autonomic function response to intervention.

**Results:**

Participants were a mean (SD) of 42.6 (10.9) years old (74% female) and 25.1 (14.1) months post-mTBI. Following 12-weeks of intervention participants had a significant improvement in symptom burden on the RPQ (i-AEP: mean change = −9.415, *p* < 0.001; d-AEP: mean change = −3.478, *p* = 0.034; c-AEP: mean change = −6.446, *p* < 0.001). Participants also had significant improvement in quality of life (i-AEP: mean change = 9.879, *p* < 0.001; d-AEP: mean change = 7.994, *p* < 0.001, c-AEP: mean change = 8.937, *p* < 0.001), dizziness (i-AEP: mean change = −11.159, *p* = 0.001; d-AEP: mean change = −6.516, *p* = 0.019; c-AEP: −8.837, *p* < 0.001) and exercise tolerance (i-AEP: mean change = 5.987, *p* < 0.001; d-AEP: mean change = 3.421, *p* < 0.001; c-AEP: mean change = 4.703, *p* < 0.001). Headache (mean change = −5.522, *p* < 0.001) and depressive symptoms (mean change = −3.032, *p* = 0.001) improved in the i-AEP group. There was no change in measures of autonomic function.

**Conclusion:**

A 12-week aerobic exercise intervention improves overall symptom burden, quality of life and specific symptom domains in adults with PPCS. Clinicians should consider prescription of progressive, individualized, sub-symptom threshold aerobic exercise for adults with PPCS even if presenting with exercise intolerance and months-to-years of symptoms.

## Introduction

Mild traumatic brain injury (mTBI) and the resulting persisting symptoms that individuals may experience places significant demands on the healthcare system and rehabilitation services. Adults who do not recover from mTBI in the expected 28 days (1, 2) may go on to experience a diverse array of symptoms grouped under the diagnosis of persisting post-concussive symptoms (PPCS) (3). PPCS commonly presents with headaches, dizziness, fatigue, low mood and sleep disturbance, in addition to exercise intolerance (4–6). Exercise intolerance, defined as worsening of post-concussive symptoms with exertion, can limit engagement in daily activities and re-incorporation of exercise into one’s routine (6, 7). In adults acutely post-injury (within 7 days) exercise intolerance has been associated with symptom burden (number of symptoms and symptom severity) (8). Fear avoidance and kinesiophobia may also play a role in delayed return to activity in those with PPCS (9–11). In adults presenting to outpatient brain injury services, the majority of individuals report not having returned to their pre-injury level of physical activity with only 28% self-reporting meeting physical activity guidelines at time of clinic presentation (12).

At present, the treatment for PPCS is largely symptom-based. While advances have been made in the treatment of PPCS, specifically pharmacologic interventions for post-traumatic headache (13, 14), few non-pharmacologic treatment modalities have shown benefit across multiple symptom domains (15, 16). In non-head injured populations, exercise has shown benefits for sleep (17–19), depression (20, 21), anxiety (22) and headache (23, 24), all of which are common post-concussive symptoms. Therefore, prescription of aerobic exercise is a non-pharmacologic, accessible intervention with the potential to play a role in PPCS rehabilitation. Pediatric studies have shown that adolescents who initiate graded, sub-symptom threshold aerobic exercise within 10 days of injury recover more quickly than those who complete a stretching program (25, 26). Based on degree of exercise tolerance, sub-symptom threshold exercise is prescribed with the goal of being tolerated by patients and not worsening symptoms. Exercise intensity can then be incrementally increased with monitoring.

The majority of past interventions evaluating exercise for rehabilitation post-mTBI have been implemented in the acute phase following injury in adolescents with sport-related concussion (25–27). Few studies have evaluated the role of prescribed exercise in rehabilitation for adults following mTBI; research thus far has either enrolled acutely concussed service members (28) or evaluated a walking intervention (29). Therefore, despite adult clinical guidelines for mTBI recovery/treatment recommending exercise (15, 30, 31), there is little evidence in adult populations with PPCS to guide clinicians in prescribing this exercise. Further, in the chronic phase of injury (>3 months post-injury) there are unique considerations for implementation of exercise interventions, such as tolerability (in those with exercise intolerance), de-conditioning and functional limitations. Here, we examine a structured, personalized 12-week aerobic exercise intervention for improvement in symptom burden for adults with PPCS with and without a precursory 6-week stretching program.

While the underlying pathophysiology of exercise intolerance is poorly understood, altered autonomic function and altered cerebral blood flow response to exertion are two possible theories (32, 33). Heart rate variability (HRV) is the beat-to-beat variation in heart rate (HR) that allows for adequate response to external stimuli (34). HRV is commonly used as a measure of autonomic function as its frequency-domain metrics approximate the contributions of the sympathetic and parasympathetic nervous system (34). Further, it is a non-invasive metric that can be measured longitudinally. Compared to age and sex-matched controls, adults with a history of mTBI have been shown to have lower HRV (35). HRV has a well established relationship with physical activity with increased activity associated with a decreased resting HR and corresponding increase in time-domain HRV (36, 37). However, the ability of aerobic exercise to modulate autonomic function in adults with PPCS has not previously been evaluated. In this study, HR, HRV and blood pressure (BP) were used to examine physiologic response to the intervention and potential for aerobic exercise to specifically improve HRV in this patient population.

The presented prospective cohort study evaluating a 12-week immediate-start aerobic exercise protocol (i-AEP) and delayed-start aerobic exercise protocol (d-AEP) aimed to: (a) evaluate change in symptom burden, quality of life (QoL) and specific post-concussive symptom outcomes; and (b) evaluate change in outcomes between those in the i-AEP and d-AEP groups.

## Materials and methods

This prospective cohort study was nested within the Aerobic Exercise for treatment of Chronic symptoms following mild Traumatic Brain Injury (ACTBI) Trial. ACTBI study methods have previously been published (38). This study was approved by the University of Calgary Conjoint Research Ethics Board (REB18-1329) and the manuscript adheres to the STROBE checklist for reporting of observational studies. The ACTBI Trial protocol was registered on clinicaltrials.gov (NCT03895450).

### Participants

Participants were recruited from outpatient brain injury, pain and physiotherapy clinics between May 2019–September 2022; recruitment was paused from March 2020–June 2021 due to COVID-19 restrictions. Inclusion criteria were: (1) adults aged 18–65 years; (2) diagnosis of mTBI (≥ 3 months to < 5 years post-injury) based on ACRM criteria (39); (3) diagnosis of PPCS based on ICD-10 criteria (40); (4) exercise intolerance (acute exacerbation of PPCS on the Buffalo Concussion Treadmill Test [BCTT] (41)) as the BCTT was used to guide exercise prescription; (5) pharmacologically stable (no change to medications, including dose, for >1 month). Exclusion criteria included: (1) neurologic diagnosis, including moderate-to-severe TBI (other than mTBI/PPCS); (2) psychiatric diagnosis (other than depression, anxiety and/or PTSD); (3) cardiopulmonary disorder (including persistent symptoms following COVID-19); (4) chronic musculoskeletal condition limiting engagement in exercise; (5) pregnancy; (6) active cancer; or (7) enrollment in another research study. Participants were cleared for physical activity by the study physician based on the Physical Activity Questionnaire for Everyone (PAR-Q+) (42).

### Intervention

For the parent study, participants were randomized to either the immediate-start AEP (i-AEP) or delayed-start AEP (d-AEP). The d-AEP group completed a 6-week stretching intervention prior to starting the 12-weeks of sub-symptom threshold aerobic exercise. Interested participants completed a phone interview to determine if they met inclusion/exclusion criteria. Following screening, if eligible and still interested in the study, participants were consented, then randomized. A computer-generated randomization sequence with block sizes of 10 was used with randomization following a 1:1 ratio. Group allocation details were contained on cards placed inside sequentially numbered, sealed, opaque envelopes. Group allocation was revealed to participants following consenting. Neither research staff/assessors nor participants were blinded to the intervention.

#### Immediate-start aerobic exercise protocol (i-AEP)

Participants completed 12-weeks of individual, sub-symptom threshold aerobic exercise at a personalized target HR. Exercise was prescribed for 5x/week for 30 min at a HR of 70–80% of the max HR achieved on the BCTT (a prescriptive treadmill test, described below) (41). A Polar H10 HR monitor (Polar Electro Oy, Kempele, Finland) was worn for the duration of activity sessions to monitor HR. The monitor had Bluetooth connectivity to a mobile application for ease of use and HR monitoring during activity. Mode and location of exercise were at the discretion of participants based on preference and access; the study team discussed potential options with participants. Exercise prescriptions were updated every 3-weeks with a repeat BCTT performed by a trained exercise physiologist and graduate student.

#### Delayed-start aerobic exercise protocol (d-AEP)

Participants randomized to the d-AEP group completed 6-weeks of stretching prior to initiation of the 12-weeks of aerobic exercise. The stretching protocol has previously been described (43). Briefly, this was a 6-week, low intensity, stretching intervention performed 30 min, 5x/week at a HR not exceeding 50% of age-predicted max HR. Results of the stretching intervention are reported elsewhere (43). Following the 6-weeks of stretching, participants went on to complete 12-weeks of aerobic exercise. They followed the same aerobic exercise intervention as the i-AEP group.

### Outcomes

All outcomes were completed at baseline and every 6-weeks thereafter. The BCTT was additionally completed every 3-weeks to update the exercise prescription and monitor exercise tolerance. Participants self-reported demographic information and injury characteristics following enrollment and randomization.

#### Clinical outcomes

The primary outcome was PPCS symptom burden on the Rivermead Post Concussion Symptoms Questionnaire (RPQ) [0–64 points] (44).

Secondary self-reported outcomes included questionnaires evaluating QoL and specific post-concussive symptoms. These included:

- QoL, measured using the Quality of Life After Brain Injury questionnaire (QOLIBRI) [0–100 points] (45, 46).
- Depressive symptoms, measured using the Patient Health Questionnaire (PHQ-9) [0–27 points] (47).
- Anxiety symptoms, measured using the Generalized Anxiety Disorder Questionnaire (GAD-7) [0–21 points] (48).
- Functional impact of headache, measured using the Headache Impact Test (HIT-6) [36–78 points] (49).
- Fatigue, measured using the Fatigue Severity Scale (FSS) [9–63 points] (50).
- Daytime sleepiness, measured using the Epworth Sleepiness Scale (ESS) [0–24 points] (51).
- Dizziness, measured using the Dizziness Handicap Index (DHI) [0–100 points] (52).

A digital daily exercise diary was completed by participants [previously described (43)]. This allowed for monitoring of adherence and participant symptom response to intervention, ensuring no significant worsening of symptoms during exercise. Any participant concerns could be noted in the diary and were followed-up by the research team.

#### Sleep assessment

Participants completed sleep monitoring for 3–6 days and nights prior to starting the exercise protocol (for the d-AEP group this was after the 6-weeks of stretching), following 6-weeks of aerobic exercise, and after 12-weeks of aerobic exercise. Participants wore a MotionWatch8 tri-axial accelerometer (CamNTech, Texas, United States) on their non-dominant wrist during this time. To supplement objective sleep actigraphy monitoring, participants completed a sleep diary every morning upon waking (53). The sleep diary was adapted from the Consensus Sleep Diary (54) and included the following questions: (a) What time did you go to bed last night?; (b) After getting into bed, how long did it take you to fall asleep?; (c) After falling asleep, how many minutes were you awake for during the night?; (d) What time did you wake up in the morning?; (e) What time did you get out of bed?; (f) How many hours did you spend in bed last night (from getting in at night, to getting out in the morning)?; (g) Did you take a nap yesterday; (h) How was the quality of your sleep last night? (1 = very poor; 5 = very good); and (i) Did you take a sleep aid or any medication to help you sleep? Sleep outcomes included sleep duration, sleep onset latency and sleep efficiency. Sleep efficiency is the total time spent asleep as a percentage of time spent in bed. Participants who had travelled >1 time zone in the past 2 months or completed a night shift in the past year did not complete sleep outcomes.

#### Exercise tolerance

Exercise tolerance (worsening of post-concussive symptoms with exertion) was assessed using the BCTT (41). The BCTT has previously been described and validated (41), but briefly, it is a progressive treadmill test where post-concussive symptom burden is rated on a 0–10 Likert scale at 1-min intervals as treadmill incline increases. HR was monitored using electrocardiogram (ECG) (CardioSoft 6.7 ECG software, GE Medical Systems, Milwaukee WI, United States) or Polar H10 HR monitor during testing. The test is terminated once the participant has a ≥ 3-point increase in symptoms. Maximum HR achieved at point of symptom exacerbation is noted. The BCTT was used to prescribe the exercise intervention as described.

#### Autonomic function assessment

Participants completed measures of autonomic function, including BP, HR and HRV at baseline and following 6-weeks and 12-weeks of intervention. All physiologic monitoring was completed under the supervision of a certified exercise physiologist.

A resting period preceded start of physiologic monitoring to establish baseline physiology. Four minutes of resting, quiet, seated HR and BP data, then 4-min of standing data were collected. Data recordings of ≥4 min have been validated for evaluating short-term HRV and shown to sufficiency approximate longer recordings (55). Between seated and standing measures, a ≥ 1 min adaptation period was included to establish resting physiology in the new posture. To ensure quiet, resting data, participants and research staff refrained from talking during testing. To address potential confounders, participants refrained from alcohol (56), caffeine (57), and vigorous exercise (58) for >6 h prior to the assessment; these have all been shown to influence HRV (59).

HR data were collected at 1000 Hz using a 12-lead ECG system, amplifier and acquisition software were used (PowerLab 16/35 amplifier, LabChart8 software, ADInstruments, Colorado Springs, CO, United States) (60). ECG was integrated with Vmax Encore 29 Metabolic Cart (Vyaire Medical, Mettawa, IL, USA). Two manual brachial BPs were taken by an exercise physiologist in both the seated and standing postures. Brachial BPs were averaged in each posture; SBP and DBP are reported.

Using ECG lead 2 from the Vmax system, PowerLab converted signal from analog-to-digital, exporting the raw ECG waveform at 1000 Hz (60). PowerLab was interfaced with LabChart to collect ECG output. ECG data was exported as a text file and imported into Ensemble-R software (V1.0.43, Ensemble, Elucimed, Wellington, NZ). ECG traces were visually inspected to identify any artifact or ectopic beats. Artifacts were replaced with interpolated RR data points using an automatic correction. Recordings with multiple ectopic beats were excluded. No pre-sets or filters were applied. Once data were normalized/cleaned, NN intervals were used to compute HRV parameters using Ensemble-R software. Time domain (SDNN, RMSSD, pNN50) and frequency domain (LF norm, HF norm, LF/HF ratio) HRV metrics are reported (34).

### Statistical analysis

Baseline sample characteristics were reported using descriptive statistics. Chi-square test, independent samples t-test or Mann–Whitney U test were used to compare i-AEP and d-AEP groups where appropriate. Sleep actigraphy data were analyzed using MotionWare1.3.17 software, which was supplemented by the sleep diary data following previously published methods to calculate the reported sleep outcomes (sleep duration, sleep onset latency, sleep efficiency) (53).

I-AEP (*n* = 27) and d-AEP (*n* = 23) groups were combined to form the combined-AEP (c-AEP; *n* = 50) group. The c-AEP group allowed for evaluation of all participants having completed the 12-week intervention.

Intention-to-treat analysis was conducted using a two-level random intercept linear regression clustering around the individual (61) to examine change in outcomes following 12-weeks of intervention for the i-AEP, d-AEP and c-AEP groups. Change in outcomes following 12-weeks of i-AEP relative to d-AEP was also examined. For each model, 95% confidence intervals were constructed using bootstrapping methods (with 1,000 repetitions) at both levels of the random intercept model helping to account for small sample size, but also minimizing the effects on inferences that potential extreme values may have had. Results with a *p*-value of <0.05 were considered statistically significant with all models adjusted for age, sex, number of previous mTBIs, months since injury, and fit with an interaction term of randomization group by time (discrete) (61). All analyses were conducted using Stata 18 (62). Mean changes with 95% confidence intervals are reported.

## Results

**Figure 1:**
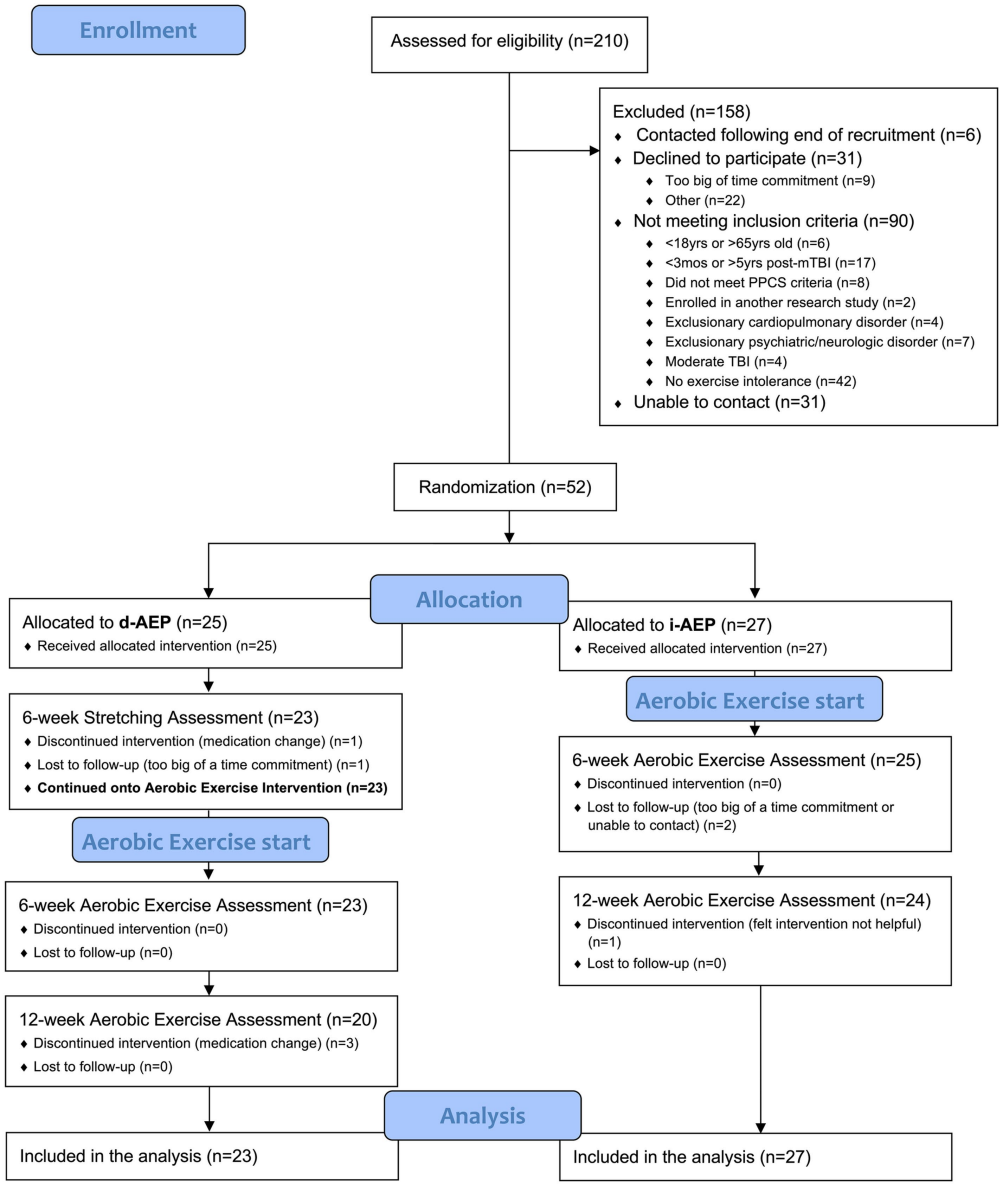
CONSORT recruitment diagram.

A total of 210 individuals were screened for the parent study. Of those, 52 individuals met inclusion criteria and agreed to participate in the parent trial. On initial randomization, 27 participants were randomized to the i-AEP and 25 participants were randomized to begin with stretching prior to d-AEP. Two participants dropped out during the stretching portion of the intervention and thus 23 participants began the d-AEP. A total of 50 participants formed the c-AEP group (*n* = 27 i-AEP; *n* = 23 d-AEP) (see Figure 1).

Over the course of the 12-weeks of exercise, a total of 6 participants dropped out. Dropouts were due to medication change (*n* = 3), loss to follow-up (*n* = 2) or felt intervention not helpful (*n* = 1).

### Demographic and injury characteristics

Participants were a mean (SD) of 42.6 (10.9) years old. It had been a mean (SD) of 25.1 (14.1) months since their most recent mTBI. Demographic characteristics including age, sex, educational status, work status and involvement in injury-related litigation did not significantly differ between the two groups. The most common mechanism of injury was motor vehicle collision (42%). See Tables 1, 2.

**Table 1:** Participant characteristics.

	c-AEP (*n* = 50)	i-AEP (*n* = 27)	d-AEP (*n* = 23)	*p* value
Age (y), mean (SD)	42.6 (10.9)	43.5 (10.5)	41.5 (11.2)	0.522
Sex, *n* (% female)	37 (74.0)	18 (66.7)	19 (82.6)	0.200 [χ^2^ = 1.641]
Education, *n* (%)				
PhD/MD/JD	1 (2.0)	1 (3.7)	0 (0.0)	0.556 [χ^2^ = 3.014]
Master’s degree	10 (20.0)	7 (25.9)	3 (13.0)	
Bachelor’s degree	21 (42.0)	11 (40.7)	10 (43.5)	
Trade school/vocational education	12 (24.0)	6 (22.2)	6 (26.1)	
Grade 12 or less	6 (12.0)	2 (7.4)	4 (17.4)	
Current work status, *n* (%)				0.138 [χ^2^ = 3.955]
Working or student	32 (64.0)	20 (74.1)	12 (52.2)	
Not currently working, but was employed or a student prior to mTBI	16 (32.0)	7 (25.9)	9 (39.1)	
Not employed/student prior to mTBI	2 (4.0)	0 (0.0)	2 (8.7)	
In litigation, *n* (% yes)	19 (38.0)	9 (33.3)	10 (43.5)	0.461 [χ^2^ = 0.543]
Medical history^†^, *n* (%)				
Endocrine	9 (18.0)	3 (11.1)	6 (26.1)	
Hypertension	4 (8.0)	2 (7.4)	2 (8.70)	
Pre-injury headache diagnosis	4 (8.0)	2 (7.41)	2 (8.70)	
Pre-injury mental health diagnosis	11 (22.0)	5 (18.5)	6 (26.1)	
Sleep disorder	4 (8.0)	3 (11.1)	1 (4.35)	
Other	28 (56.0)	16 (59.3)	12 (52.2)	
Medications, *n* (%)				
Anti-depressant/anti-psychotic/neurostimulant	27 (54.0)	14 (51.9)	13 (56.5)	
Anti-epileptic	4 (8.0)	2 (7.4)	2 (8.7)	
Anti-nausea/GI	9 (18.0)	4 (14.8)	5 (21.7)	
Cardiovascular	3 (6.0)	2 (7.4)	1 (4.3)	
Endocrine	7 (14.0)	2 (7.4)	5 (21.7)	
Headache (preventative/abortive)	10 (20.0)	8 (29.6)	2 (8.7)	
HRT	2 (4.0)	0 (0.0)	2 (8.7)	
Metabolic	4 (8.0)	3 (11.1)	1 (4.3)	
Pain	14 (28.0)	6 (22.2)	8 (34.8)	
Respiratory/anti-histamine	4 (8.0)	3 (11.1)	1 (4.3)	
Sleep	16 (32.0)	9 (33.3)	7 (30.4)	
Pre-intervention questionnaires, mean (SD)				
RPQ [0–64]	33.2 (10.6)	34.9 (8.3)	31.2 (12.6)	0.229
QOLIBRI [0–100]	49.5 (13.4)	51.8 (12.4)	46.9 (14.2)	0.199
PHQ-9 [0–27]	12.6 (5.2)	12.5 (5.8)	12.7 (4.3)	0.905
GAD-7 [0–21]	7.3 (5.2)	6.9 (5.2)	7.7 (5.3)	0.625
HIT-6 [36–78]	61.1 (6.6)	63.0 (6.0)	58.8 (6.7)	0.026*
FSS [9–63]	46.1 (1.8)	45.6 (13.0)	46.7 (12.6)	0.763
ESS [0–24]	8.0 (4.6)	8.0 (4.7)	8.0 (4.6)	0.974
DHI [0–100]	34.8 (24.7)	32.6 (23.1)	37.3 (26.7)	0.592

c-AEP, combined aerobic exercise protocol; d-AEP, delayed-start aerobic exercise protocol; DHI, Dizziness Handicap Inventory; ESS, Epworth Sleepiness Scale; FSS, Fatigue Severity Scale; GAD-7, Generalized Anxiety Disorder Scale; GI, gastrointestinal; HIT-6, Headache Impact Test; HRT, hormone replacement therapy; i-AEP, immediate-start aerobic exercise protocol; JD, Juris Doctor; MD, Doctor of Medicine; mTBI, mild traumatic brain injury; PhD, Doctor of Philosophy; PHQ-9, Patient Health Questionnaire; RPQ, Rivermead Post Concussion Symptoms Questionnaire; QOLIBRI, Quality of Life After Brain Injury Questionnaire. *Statistically significant at *p* < 0.05. † At time of starting intervention; endocrine and hypertension pre and post-injury.

**Table 2:** Injury characteristics, physical activity and exercise testing.

	c-AEP (*n* = 50)	i-AEP (*n* = 27)	d-AEP (*n* = 23)	*p* value
Injury characteristics				
Months since injury, mean (SD)	25.1 (14.1)	25.3 (12.9)	24.8 (15.7)	0.900
Mechanism of injury, n (%)				0.127 [χ^2^ = 8.573]
Sports/recreation	15 (30.0)	9 (33.3)	6 (26.1)	
Fall	7 (14.0)	6 (22.2)	1 (4.3)	
Work-related	2 (4.0)	2 (7.4)	0 (0.0)	
MVC	21 (42.0)	7 (25.9)	14 (60.9)	
Assault	3 (6.0)	2 (7.4)	1 (4.3)	
Other	2 (4.0)	1 (3.7)	1 (4.3)	
Loss of consciousness, n (%)				0.478 [χ^2^ = 1.475]
Yes	14 (28.0)	9 (33.3)	5 (21.7)	
No	28 (56.0)	13 (48.1)	15 (65.2)	
Unknown	8 (16.0)	5 (18.5)	3 (13.0)	
Post-traumatic amnesia, *n* (%)				0.187 [χ^2^ = 3.359]
Yes	21 (42.0)	13 (48.1)	8 (34.8)	
No	24 (48.0)	10 (37.0)	14 (60.9)	
Unknown	5 (10.0)	4 (14.8)	1 (4.3)	
Lifetime mTBI, *n* (%)				0.461 [χ^2^ = 0.543]
1	19 (38.0)	9 (33.3)	10 (43.5)	
≥ 2	31 (62.0)	18 (66.7)	13 (56.5)	
Pre-injury participation in sport^†^, *n* (% yes)	15 (30.0)	9 (33.3)	6 (26.1)	0.577 [χ^2^ = 0.311]
Pre-intervention exercise test, mean (SD)				
Resting seated HR (bpm)	75.1 (10.1)	73.0 (9.0)	77.4 (10.9)	0.137
Resting seated systolic BP (mmHg)	113.7 (12.7)	117.2 (13.0)	109.7 (11.2)	0.034*
Resting seated diastolic BP (mmHg)	74.0 (7.8)	77.1 (5.7)	70.3 (8.4)	0.001*
Pre-test symptom score^‡|^[0–10]	1.8 (1.3)	1.7 (1.4)	1.9 (1.3)	0.620
HR at symptom exacerbation on BCTT (bpm)	136.7 (23.3)	137.1 (23.5)	136.3 (23.6)	0.914
Highest BCTT stage reached	10.0 (4.5)	9.7 (4.9)	10.3 (4.1)	0.687

BCTT, Buffalo Concussion Treadmill Test; bpm, beats per minute; BP, blood pressure; c-AEP, combined aerobic exercise protocol; d-AEP, delayed-start aerobic exercise protocol; HR, heart rate; i-AEP, immediate-start aerobic exercise protocol; mmHg, millimeters of mercury; mTBI, mild traumatic brain injury; MVC, motor vehicle collision. * Statistically significant at *p* < 0.05. † Response to question, “At the time of your brain injury were you involved in organized or competitive athletics?” ‡ Symptoms on Likert scale at start of BCTT.

Groups did not significantly differ in symptom burden nor specific symptom outcomes pre-intervention with the exception of functional impact of headache. HIT-6 scores were significantly higher (*p* = 0.026) in i-AEP compared to d-AEP pre-intervention (see Table 1).

### Clinical outcomes—symptoms, QoL, sleep

Following 12-weeks of intervention, overall participants had a significant improvement in symptom burden on the RPQ (i-AEP: mean change = −9.415, *p* < 0.001; d-AEP: mean change = −3.478, *p* = 0.034; c-AEP: mean change = −6.446, *p* < 0.001). The i-AEP group had a significantly greater improvement compared to the d-AEP group on the RPQ. When looking at all participants (c-AEP), the improvement on the RPQ exceeded the minimum clinically important difference of 4.6 points (63).

Participants also had significant improvement in QoL on the QOLIBRI (i-AEP: mean change = 9.879, *p* < 0.001; d-AEP: mean change = 7.994, *p* < 0.001, c-AEP: mean change = 8.937, *p* < 0.001). Improvement in QoL did not significantly differ between the two groups (i-AEP, d-AEP).

Following intervention there was improvement in specific post-concussive symptoms, including dizziness (i-AEP: mean change = −11.159, *p* = 0.001; d-AEP: mean change = −6.516, *p* = 0.019; c-AEP: −8.837, *p* < 0.001) and exercise tolerance (i-AEP: mean change = 5.987, *p* < 0.001; d-AEP: mean change = 3.421, *p* < 0.001; c-AEP: mean change = 4.703, *p* < 0.001). Headache (mean change = −5.522, *p* < 0.001), depression (mean change = −3.032, *p* = 0.001) and fatigue (mean change = −9.047, *p* < 0.001) improved in the i-AEP group.

**Figure 2:**
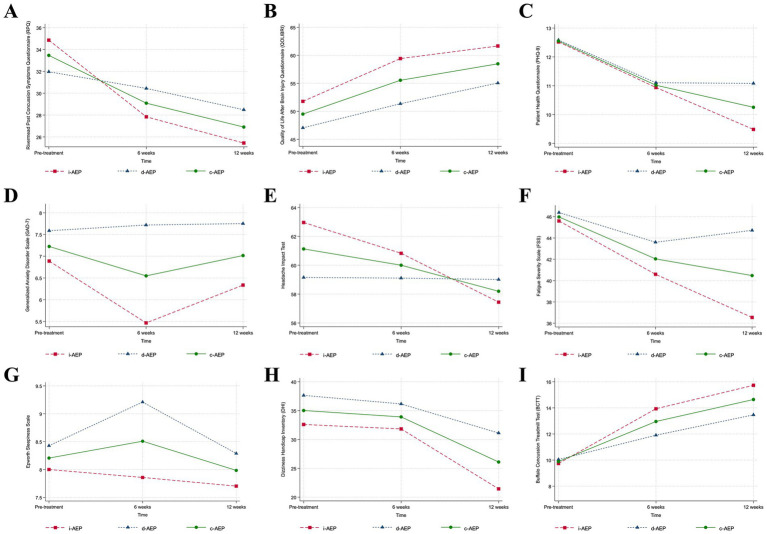
Clinical outcomes. Conditions are i-AEP (immediate-start aerobic exercise protocol; *n* = 27), d-AEP (delayed-start aerobic exercise protocol; *n* = 23) and c-AEP (combined aerobic exercise protocol; *n* = 50). Mean change from pre-treatment to 12-weeks of intervention from linear mixed-effect model for **(A)** symptom burden (Rivermead Post Concussion Symptoms Questionnaire, RPQ [0–64]); **(B)** quality of life (Quality of Life After Brain Injury Questionnaire, QOLIBRI [0–100]); **(C)** depressive symptoms (Patient Health Questionnaire-9, PHQ-9 [0–27]); **(D)** anxiety symptoms (Generalized Anxiety Disorder Scale, GAD-7 [0–21]); **(E)** functional impact of headache (Headache Impact Test, HIT-6 [36-78]); **(F)** fatigue (Fatigue Severity Scale, FSS [9-63]); **(G)** daytime sleepiness (Epworth Sleepiness Scale, ESS [0–24]); **(H)** dizziness (Dizziness Handicap Index, DHI [0–100]); **(I)** exercise intolerance (Buffalo Concussion Treadmill Test, BCTT).

There was no significant change in sleep outcomes (sleep duration, sleep onset latency, sleep efficiency) for either group across 12-weeks of intervention. Results are reported in Table 3 and Figure 2.

**Table 3:** Clinical outcomes.

	i-AEP (*n* = 27)			d-AEP (*n* = 23)			Between group difference			c-AEP (*n* = 50)		
Clinical outcome	Mean change	(95% CI)	*p* value	Mean change	(95% CI)	*p* value	Mean change	(95% CI)	*p* value	Mean change	(95% CI)	*p* value
RPQ [0–64]	−9.415	(−14.113, −4.717)	<0.001*	−3.478	(−6.712, −0.254)	0.034*	−5.937	(−11.657, −0.227)	0.042*	−6.446	(−9.289, −3.604)	<0.001*
QOLIBRI [0–100]	9.879	(5.561, 14.198)	<0.001*	7.994	(4.305, 11.683)	<0.001*	1.885	(−3.839, 7.610)	0.519	8.937	(6.119, 11.754)	<0.001*
PHQ-9 [0–27]	−3.032	(−4.853, −1.212)	0.001*	−1.501	(−3.056, 0.055)	0.059	−1.532	(−3.934, 0.871)	0.211	−2.266	(−3.460, −1.073)	<0.001*
GAD-7 [0–21]	−0.553	(−2.360, 1.254)	0.549	0.163	(−1.383, 1.710)	0.836	−0.717	(−3.044, 1.611)	0.546	−0.195	(−1.410, 1.020)	0.753
HIT-6 [36–78]	−5.522	(−8.235, −2.808)	<0.001*	−0.142	(−2.309, 2.026)	0.898	−5.380	(−8.790, −1.970)	0.002*	−2.832	(−4.600, −1.064)	0.002*
FSS [9–63]	−9.047	(−14.120, −3.975)	<0.001*	−1.668	(−4.515, 1.179)	0.251	−7.379	(−13.218, −1.541)	0.013*	−5.358	(−8.255, −2.460)	<0.001*
ESS [0–24]	−0.297	(−1.337, 0.742)	0.575	−0.139	(−1.412, 1.134)	0.831	−0.159	(−1.832, 1.514)	0.853	−0.218	(−1.025, 0.589)	0.596
DHI [0–100]	−11.159	(−17.794, −4.523)	0.001*	−6.516	(−11.941, −1.091)	0.019*	−4.642	(−13.316, 4.031)	0.294	−8.837	(−13.071, −4.604)	<0.001*
BCTT stage	5.987	(4.039, 7.935)	<0.001*	3.421	(1.988, 4.853)	<0.001*	2.566	(0.178, 4.955)	0.035*	4.703	(3.480, 5.928)	<0.001*
Sleep duration (hours)	0.112	(−0.161, 0.385)	0.421	−0.068	(−0.402, 0.265)	0.688	0.180	(−0.241, 0.602)	0.402	0.021	(−0.198, 0.242)	0.846
Sleep onset latency (minutes)	−2.677	(−7.645, 2.291)	0.291	2.969	(−6.955, 12.893)	0.558	−5.646	(−16.963, 5.670)	0.328	0.145	(−5.291, 5.583)	0.958
Sleep efficiency (%)	0.832	(−0.486, 2.150)	0.216	−1.635	(−3.626, 0.357)	0.108	2.467	(0.014, 4.920)	0.049*	−0.401	(−1.563, 0.760)	0.498

Mean change and 95% confidence intervals from the random intercept model adjusted for age, sex, number of previous mTBIs and months since injury of clinical outcomes from pre-treatment to 12-weeks of intervention. BCTT, Buffalo Concussion Treadmill Test; c-AEP, combined aerobic exercise protocol; d-AEP, delayed-start aerobic exercise protocol; DHI, Dizziness Handicap Inventory; ESS, Epworth Sleepiness Scale; FSS, Fatigue Severity Scale; GAD-7, Generalized Anxiety Disorder Scale; HIT-6, Headache Impact Test; i-AEP, immediate-start aerobic exercise protocol; PHQ-9, Patient Health Questionnaire; RPQ, Rivermead Post Concussion Symptoms Questionnaire; QOLIBRI, Quality of Life After Brain Injury Questionnaire. * Statistically significant at *p* < 0.05.

### Physiologic outcomes—HR, BP, HRV

There were no significant changes in seated or standing HR, BP or HRV metrics following 12-weeks of intervention in either the i-AEP, d-AEP or c-AEP group. Results did not significantly differ between i-AEP and d-AEP. Results of c-AEP group are reported in Table 4.

**Table 4:** Resting seated and standing heart rate variability and blood pressure.

	Mean change	(95% CI)	*p* value
Seated posture			
c-AEP, pre-intervention to 12-weeks (*n* = 50)			
HR (bpm)	0.457	(−1.808, 2.000)	0.692
pNN50 (%)	0.071	(−3.379, 3.523)	0.967
RMSSD (ms)	−0.863	(−4.900, 3.174)	0.675
SDNN (ms)	−2.373	(−6.613, 1.865)	0.272
LF norm (n.u.)	−1.548	(−6.547, 3.452)	0.544
HF norm (n.u.)	1.548	(−3.282, 6.377)	0.530
LF/HF	−0.877	(−2.268, 0.513)	0.216
SBP^†^ (mmHg)	−2.478	(−5.005, 0.049)	0.055
DBP^†^ (mmHg)	−1.048	(−2.860, 0.765)	0.257
Standing posture			
c-AEP, pre-treatment to 12-weeks (*n* = 50)			
HR (bpm)	0.825	(−2.000, 3.650)	0.567
pNN50 (%)	−0.472	(−2.213, 1.268)	0.595
RMSSD (ms)	−0.364	(−3.233, 2.504)	0.803
SDNN (ms)	0.337	(−4.582, 5.256)	0.893
LF norm (n.u.)	−0.778	(−4.330, 2.775)	0.668
HF norm (n.u.)	0.778	(−2.740, 4.296)	0.665
LF/HF	−0.917	(−2.000, 0.164)	0.096
SBP^†^ (mmHg)	0.267	(−2.756, 3.290)	0.863
DBP^†^ (mmHg)	−0.935	(−2.684, 0.813)	0.294

Mean change and 95% confidence intervals from linear mixed-effect model of clinical outcomes from pre-treatment to 12-weeks of intervention for c-AEP (*n* = 50). c-AEP, combined aerobic exercise protocol; DBP, diastolic blood pressure; HR, heart rate; HF norm, high frequency power in normalized units; LF norm, low frequency power in normalized units; LF/HF ratio, low frequency to high frequency ratio; pNN50, percent of RR intervals differing by more than 50 ms; RMSSD, root mean squared of successive RR interval differences; SBP, systolic blood pressure; SDNN, standard deviation of RR intervals. † Average of two manual brachial blood pressures.

### Adverse events

There were no reported adverse events related to the intervention itself that occurred during either completion of i-AEP, d-AEP nor during exercise testing.

## Discussion

This prospective cohort study evaluating a 12-week sub-symptom threshold aerobic exercise intervention for adults with PPCS is an important first step in providing data to support the prescription of aerobic exercise for this specific patient population. While aerobic exercise is increasingly being recognized for its important role in rehabilitation following mTBI in adolescent and athletic populations, studies evaluating the impact of exercise for adults with persisting symptoms following injury are lacking (27, 64). Here, we demonstrated that among participants who completed a 12-week exercise intervention (c-AEP; *n* = 50), there was a significant improvement in overall symptom burden, QoL and specific symptom domains (depressive symptoms, headache, dizziness, exercise tolerance). Individuals who completed a precursory 6-week stretching program (d-AEP group) did not have a greater response to exercise compared to those who engaged in the exercise immediately following enrollment (i-AEP group). This trial suggests that adults presenting with months-to-years of symptoms following mTBI can both tolerate and benefit from a graded, personalized aerobic exercise intervention. While altered autonomic function has been recognized in cohorts with mTBI and PPCS (32, 65), the described intervention did not change resting HR, BP nor measures of HRV. While further study of the mechanisms by which exercise may improve post-concussive symptoms and exercise tolerance is needed, this work provides preliminary data to support prescription of personalized HR-targeted aerobic exercise for adults with PPCS.

Landmark trials evaluating exercise prescription following adolescent sport-related concussion found that initiation of a sub-symptom threshold aerobic exercise program within 10 days of injury could speed recovery and lead to a reduced incidence of PPCS compared to stretching (25, 26). Similarly, an exploratory RCT in an adolescent cohort with 4–16 weeks of PPCS also showed greater improvement in symptom burden in those who completed a 7-week aerobic exercise program compared to stretching (66). Several other studies, in both adults and adolescents, have investigated rehabilitation programs with an exercise component and shown benefit, but where evaluation of a structured exercise intervention was not the primary aim (64). In this study we demonstrated that adults with months-to-years of PPCS (c-AEP; *n* = 50) who completed 12-weeks of tailored, sub-symptom threshold aerobic exercise had a significant improvement in post-concussive symptoms as measured on the RPQ. This improvement (c-AEP group; mean change = −6.446) exceeded the suggested minimal clinically important difference. The RPQ measure of symptom burden is composed of 16-items representing the most common post-concussive symptoms and therefore is a clinically relevant measure of symptom improvement over time. Further, specific symptom questionnaires were useful in identifying symptoms that were most responsive to the intervention. When looking at all participants (c-AEP), there was significant improvement in depressive symptoms, functional impact of headache, fatigue and dizziness over time. Aerobic exercise has been well documented to improve many of these same clinical symptoms in non-head injured populations, specifically depression (21) and headache (23, 24); this trial suggests similar benefits for individuals with PPCS.

On the most comprehensive self-reported outcome, QoL measured using the QOLIBRI questionnaire, both groups (d-AEP; i-AEP) had a significant improvement in their scores across the intervention. Further, the d-AEP and i-AEP had a similar improvement in QoL over time, suggesting that initiation of a stretching program prior to exercise does not confer additional exercise benefit. While no minimal clinically important difference is available for the QOLIBRI, the 8.9 point improvement (out of 100 points) for the c-AEP group, combined with improvements to exercise tolerance and multiple other symptoms suggests QoL improvement was not only statistically, but also functionally significant. A significant improvement on the QOLIBRI was also found following an 8-week walking intervention in adults with PPCS (29). Improvements in QoL have also been reported in a cohort with all severity TBI, where those who reported exercising ≥ 90 min/week had higher QoL compared to those who did not in a secondary analysis (67). The mechanism by which exercise improves QoL in this patient population was beyond the scope of this study; however, increased self-efficacy, a return to an important pre-morbid activity (30% of participants were engaged in sports at the time of injury) and increased socialization (many participants reported completing the activity with a partner or friend) may all have contributed.

I-AEP and d-AEP groups both had improvements in symptom burden and measures of specific post-concussive symptoms. On some outcomes, including symptom burden, functional impact of headache, fatigue, exercise tolerance and sleep efficiency, the i-AEP group had a significantly greater improvement compared d-AEP. Given the groups were relatively well matched it is unclear the exact reason for these differences in response; however, we propose several factors contributed. In terms of symptom burden, the significantly greater improvement in the i-AEP group may have been in part driven by the d-AEP group’s completion of the precursory stretching program. While the stretching lead to a non-significant improvement in symptom burden (43), it is possible that this initial improvement in symptoms resulted from the additional weeks of interaction that the d-AEP group had with the study team ahead of starting the 12-week aerobic protocol and having already been primed to the study protocol. By randomizing to an arm with 6 weeks of stretching first (d-AEP) we were able to evaluate the influence of the placebo effect on our clinical outcomes, an important consideration in any randomized clinical trial. Difference in functional outcome of headache response (significantly greater improvement in the i-AEP vs. d-AEP) may have been in part due to between group differences at baseline, where the i-AEP group had higher headache scores compared to d-AEP. Differences in sleep efficiency response between groups was driven by the d-AEP group, which decreased by 1.6%, whereas the i-AEP group had a marginal improvement in sleep efficiency of 0.8%. There is no minimal clinical importance difference threshold for sleep efficiency based on actigraphy in PPCS to our knowledge. However, studies showing significant improvement in sleep efficiency following cognitive behavioural therapy for insomnia (gold standard intervention) in adults with cardiovascular disease showed a mean improvement in sleep efficiency (measured using actigraphy) of 0.5–16%, suggesting that the marginal changes to sleep efficiency in this study are likely not of clinical significance (68–71).

Aerobic exercise has been suggested as a treatment modality to improve autonomic function following mTBI (72). Following 12-weeks of intervention we found no significant change in HR, HRV or BP in either the seated or standing posture. Several factors likely contributed to this null result, including intervention intensity, duration and participant age. At the start of the exercise intervention, participants presented with a high degree of exercise intolerance, meaning they were able to complete relatively few stages on the BCTT prior to symptom worsening. Given this, exercise prescriptions were of relatively low intensity at initiation of intervention. As participants progressed through the study, exercise prescriptions were updated in accordance with results on the BCTT. However, many participants still did not reach their age-predicted max HR on treadmill testing without becoming symptomatic at the final assessment. This suggests the intervention may not have been of sufficient duration to fully return individuals to age-predicted level of exercise. Several previous studies have shown that physical activity volume and intensity are associated with HRV metrics (37, 73, 74). In a community-based study of 985 older adults Soares-Miranda et al. demonstrated that higher total leisure-time activity, walking distance and walking pace were all positively associated with 24-h SDNN (73). These results were built upon by de Sousa et al. who evaluated association between resting HRV measures and amount of very vigorous physical activity measured via hip accelerometry (75). All HRV variables (including SDNN and RMSSD) demonstrated a linear trend through the quintiles of very vigorous physical activity; those who completed a greater duration of very vigorous physical activity had higher time-domain HRV metrics (75). Age also likely plays a role in HRV response to physical activity. HRV data estimated by photoplethysmography has suggested a greater volume of activity is required to see HRV changes with increasing age, which could be another contributing factor in this study (37). Using photoplethysmography data Natarajan et al. suggested that individuals aged 20–24 years could increase their HF power by 1ms^2^ with an additional ~30 steps/day, but that 200–300 steps/day would be needed to see the same improvement in those aged 50–54 years (37). Taken together, these results suggest that a longer intervention with greater time to build exercise tolerance, and thus allow for engagement in more vigorous exercise, is likely required to observe improvement in HRV parameters in adults with PPCS. Future work may consider inclusion of additional parameters of autonomic function to further understand physiologic response to exercise interventions in adults with PPCS.

### Limitations

There were limitations to this study, notably the lack of a control group for the duration of the 12-weeks of exercise. While significant improvement was observed over the course of the intervention, a longer intervention would likely have provided additional insights into symptom change over time with exercise. A longer follow-up period would have also allowed more time for participants to build up their exercise tolerance and thus engage in more moderate-to-vigorous prescribed exercise. This prospective cohort study was conducted at a single center and recruited from specialized brain injury and pain clinics; therefore, the results are generalizable to individuals with PPCS presenting to specialized care. Multi-centered trials across multiple sites with broader recruitment are needed to fully understand generalizability of such an intervention for adults with PPCS. A greater proportion of females (74%) compared to males (26%) were enrolled in the study. This may have been in part due to the fact that more females than males go on to experience persisting symptoms following mTBI, or that women are more likely to seek care (76, 77). Regardless, this too has implications for generalizability. In terms of autonomic testing, resting HR, BP and HRV measures were completed. In future, additional measures of autonomic function (including assessments such as facial cooling, valsalva maneuver or baroreceptor sensitivy) might reveal more subtle alterations in autonomic function that were not appreciated in this study.

### Clinical applicability and future directions

Previous reviews of treatments for PPCS have provided the guidance to “consider offering graded physical exercise in addition to other treatment” despite a lack of evidence for how this exercise should be prescribed, its benefits and tolerability (15). Here, we present an exercise intervention with symptom and QoL benefits that could be incorporated into the multi-disciplinary care of adults with PPCS. Given the exercise intervention was well tolerated, without adverse events, it presents a low-cost, non-pharmacologic, personalized intervention that could be prescribed without fear of exacerbating symptoms so long as adequate monitoring is in place. Participants largely found the HR monitoring using an HR monitor to be helpful for compliance during independent exercise sessions. Currently, there are few clinical settings where this type of exercise testing (BCTT) and subsequent exercise prescription is available for patients with PPCS. Other groups have published methods for prescribing HR-targeted exercise based on age-predicted HR maximum, which may be considered when formal exercise testing is not available (78, 79). Additionally, more work is needed in this area to better understand which patients may preferentially respond to this type of intervention, the optimal duration of intervention, and adherence from participants upon study termination (long follow-up timelines). Given the intervention was well tolerated, in select patient we suggest prescription of activity at 80–90% of max HR achieved on the BCTT could be trialed (instead of the 70–80% used in this study).

## Conclusion

To date, few studies have evaluated the prescription of sub-symptom threshold aerobic exercise in adult PPCS cohorts and thus limited data are available to treating clinicians in terms of prescribing such an intervention. We found that following 12-weeks of sub-symptom threshold aerobic exercise, participants (c-AEP; n = 50) had significant improvement in symptom burden, QoL and specific symptom domains. Further, there was improvement in exercise tolerance enabling engagement in more vigorous exercise over time. This provides preliminary data to support prescription of individualized, sub-symptom threshold aerobic exercise for adults with PPCS following a graduated approach of increasing intensity based on exercise tolerance.

## Data Availability

The datasets presented in this article are not readily available because a formal data sharing agreement would have to be initiated by any researchers requesting this data set. Requests to access the datasets should be directed to Leah Mercier, leah.mercier@ucalgary.ca.
